# Interpreting COVID-19 and Virtual Care Trends: Cohort Study

**DOI:** 10.2196/18811

**Published:** 2020-04-15

**Authors:** Saif Khairat, Chenlu Meng, Yuxuan Xu, Barbara Edson, Robert Gianforcaro

**Affiliations:** 1 School or Nursing University of North Carolina at Chapel Hill NC, NC United States; 2 School of Information and Library Science University of North Carolina at Chapel Hill Chapel Hill, NC United States; 3 UNC Health Chapel Hill, NC United States

**Keywords:** virtual care, COVID-19, trends, patterns, pandemic, outbreak, infectious disease, public health

## Abstract

**Background:**

The coronavirus disease (COVID-19) pandemic is rapidly spreading across the world. As of March 26, 2020, there are more than 500,000 cases and more than 25,000 deaths related to COVID-19, and the numbers are increasing by the hour.

**Objective:**

The aim of this study was to explore the trends in confirmed COVID-19 cases in North Carolina, and to understand patterns in virtual visits related to symptoms of COVID-19.

**Methods:**

We conducted a cohort study of confirmed COVID-19 cases and patients using an on-demand, statewide virtual urgent care center. We collected data from February 1, 2020, to March 15, 2020. Institutional Review Board exemption was obtained prior to the study.

**Results:**

As of March, 18 2020, there were 92 confirmed COVID-19 cases and 733 total virtual visits. Of the total visits, 257 (35.1%) were related to COVID-19-like symptoms. Of the COVID-19-like visits, the number of females was 178 (69.2%). People in the age groups of 30-39 years (n=67, 26.1%) and 40-49 years (n=64, 24.9%) were half of the total patients. Additionally, approximately 96.9% (n=249) of the COVID-like encounters came from within the state of North Carolina. Our study shows that virtual care can provide efficient triaging in the counties with the highest number of COVID-19 cases. We also confirmed that the largest spread of the disease occurs in areas with a high population density as well as in areas with major airports.

**Conclusions:**

The use of virtual care presents promising potential in the fight against COVID-19. Virtual care is capable of reducing emergency room visits, conserving health care resources, and avoiding the spread of COVID-19 by treating patients remotely. We call for further adoption of virtual care by health systems across the United States and the world during the COVID-19 pandemic.

## Introduction

The coronavirus disease (COVID-19) pandemic is rapidly spreading across the world. As of March 26, 2020, there were more than 500,000 cases and more than 25,000 deaths related to COVID-19, and the numbers continue to increase [1,2]. The swift transmission of COVID-19 is a threat to the world. It hinders our ability to contain the spread or the damage [3]. Many countries restricted air travel in and out of the country in an attempt to stop, or at least slow down, the transmission of the disease. However, the numbers of infected people are in an exponential and rapid growth [4].

Calls were made to promote and use virtual care (VC) such as remote medical consultations as an effort toward enforcing social distancing, using resources efficiently, and improving health care access [5]. The US government and private payers such as insurance companies have been working closely to remove any restrictions on the use of VC, also known as telehealth [6]. Now people can use consumer applications such as FaceTime, Google Hangout, and other video chat platforms to interact with a medical provider remotely [7]. Additionally, insurance coverage now covers VC between a provider and patient who have not met in person. All these attempts were necessary to avoid the gathering of large numbers of people in the same space, except for medical reasons [8]. In this study, we explored possible trends in confirmed COVID-19 cases along with COVID-19-like virtual visits. We hypothesized that there was a pattern between the location and duration of the confirmed COVID-19 cases and COVID-19-like virtual calls prior to the occurrence of confirmed cases.

The aim of this study was to explore the trends in confirmed COVID-19 cases in North Carolina, and to understand patterns in virtual visits related to symptoms of COVID-19.

## Methods

### Virtual Urgent Care

We conducted a cohort study of confirmed COVID-19 cases and patients using an on-demand, statewide virtual urgent care (VUC) center. The center was launched by a major health care system in the Southeast region of the United States. We collected data from February 1, 2020, to March 15, 2020. Institutional Review Board exemption was obtained prior to the study.

Our choice of study start date being February 1, 2020 stems from the first COVID-19 case in the United States, which occurred in Washington state on January 21, 2020. The first case in North Carolina was March 3, 2020 related to a person traveling from Washington state. This indicates that during the month of February there was active transmission of COVID-19 across the United States that we did not know about due to a lack of screening and testing.

### Data Sources

We collected data during the selected dates using the numbers of confirmed COVID-19 cases reported by the North Carolina Department of Health and Human Services (NCDHHS). Additionally, we analyzed COVID-19-like virtual visits from February 1 to 28, 2020, prior to the first confirmed COVID-19 case (March 3, 2020).

### Analysis for Virtual Visits With COVID-19-Like Symptoms

The VC data included patient demographics and chief complaints. We stratified the virtual visits into two groups: COVID-19-like visit and all other visits. We categorized a virtual visit as a COVID-19-like visit if the chief complaint mentioned by the patient overlapped with COVID-19 symptoms reported by the Centers for Disease Control and Prevention and the World Health Organization (WHO), such as “cough,” “fever,” “respiratory infection,” or “fatigue” [9,10]. Throughout the paper, we will use the term “COVID-19 like” to refer to virtual visits where patients reported chief complaints that were similar to COVID-19. At the time of the study, there were no virtual COVID-19 tests to screen if these virtual visits had patients who were indeed COVID-19 carriers.

### Analysis for COVID-19 Confirmed Cases

Based on information we retrieved from the NCDHHS, we mapped the number of confirmed cases on a North Carolina map. Then, we identified the major areas of attraction within the counties with the highest concentrations of confirmed COVID-19 cases to rationalize the reasons for the high concentration of cases. Additionally, we ran a time-motion analysis on the confirmed cases over time to understand the duration and the extent of disease spread across the state counties. To demonstrate the time-motion of the disease, we used a color palette such that each color represents confirmed COVID-19 cases on a given date between March 3-18, 2020.

To plot the confirmed COVID-19 cases in North Carolina geographically, we created a map showing the number of cases for each county and assigned the color shades according to the number of cases, the deeper the color, the more cases. Furthermore, to explore the relationships between VUC COVID-19-like encounters and confirmed COVID-19 cases, we labeled the encounters as “two weeks before the outbreak” (encounters in February 2020) and “after the outbreak” (encounters from March 1 to March 15, 2020). Then, the number of encounters of each county were displayed geographically on a map based on the same rule as the map of COVID-19 cases for a straightforward concept and comparison. To delve into the trend of how local COVID-19 cases increase temporally and spatially, we divided the COVID-19 cases using 2 time ranges: a 7-day time range and a day-by-day time range. A bar chart and a pie chart on the map were created based on the broken-down data.

### Data Analysis

We analyzed patient demographics of the COVID-19-related virtual visits. We conducted exploratory analysis based on their gender, age group, and state of residence. Since all visits happened through phone calls or video calls, it was important to use patients’ state of residence to analyze their characteristics. Certain tools such as Microsoft Excel were used throughout this process and to display the results. All the data processing work was conducted in Python (Python Software Foundation) using NumPy and Pandas library and the visualizations were created using Tableau Software. This was beneficial to detect any trend or patterns of patients’ behaviors.

## Results

### Analysis of Confirmed Cases

As of March, 18 2020, there were 92 confirmed COVID-19 cases and 733 total virtual visits. Of the total visits, 257 (35.1%) were related to COVID-19-like symptoms. Of the COVID-19-like visits, nearly three-fourths were female. People in the age groups 30-39 years and 40-49 years were half of the total patients. Additionally, almost all COVID-like encounters came from within the state of North Carolina (Table 1).

**Table 1:** Summary of Characteristics of Virtual Care Patients with COVID-19 Symptoms (N=257).

Virtual care demographics		Encounters, n (%)
**Gender**		
	Female	178 (69.3)
	Male	75 (29.2)
	Nonbinary	4 (1.6)
**Age group (years)**		
	<10	28 (10.9)
	10-20	23 (8.9)
	20-30	40 (15.6)
	30-40	67 (26.1)
	40-50	61 (23.7)
	50-60	31 (12.1)
	60-70	7 (2.7)
**State of residence**		
	Florida	1 (0.4)
	Georgia	1 (0.4)
	North Carolina	249 (96.9)
	New Jersey	1 (0.4)
	South Carolina	2 (0.8)
	Virginia	2 (0.8)
	Wisconsin	1 (0.4)

North Carolina, like other US states, observed an increase in COVID-19 confirmed cases in a short time period [11]. The first case was recorded on March 3, 2020; in 10 days, the number of cases escaladed to 24 cases, and then, in only 3 days, there was a steep increase to 64 cases to reach a total of 92 confirmed COVID-19 cases by March 18, 2020.

**Figure 1:**
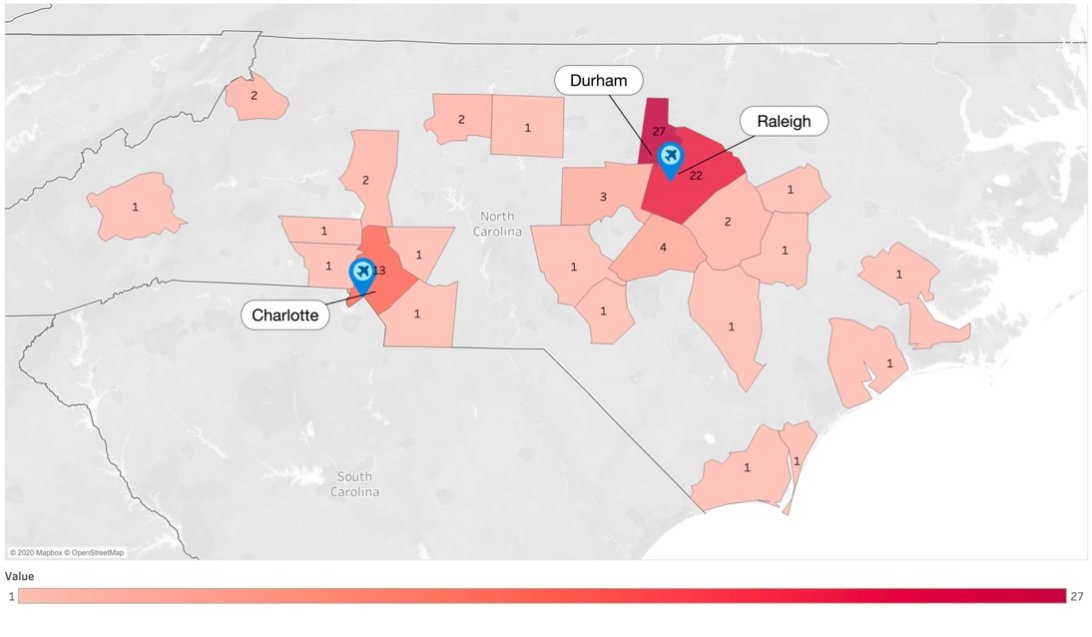
Mapping of North Carolina confirmed coronavirus disease cases with airport locations.

The North Carolina map shown in Figure 1 shows that 62 (67%) of the 92 COVID-19 confirmed cases occurred within two counties with the highest density in the state and house two major international airports in North Carolina, namely Raleigh-Durham International Airport (RDU) and Charlotte Douglas International Airport (CLT). Figure 1 also shows that there are scattered individual cases of COVID-19 in the eastern and southwestern parts of the state, which are typically less dense regions.

**Figure 2:**
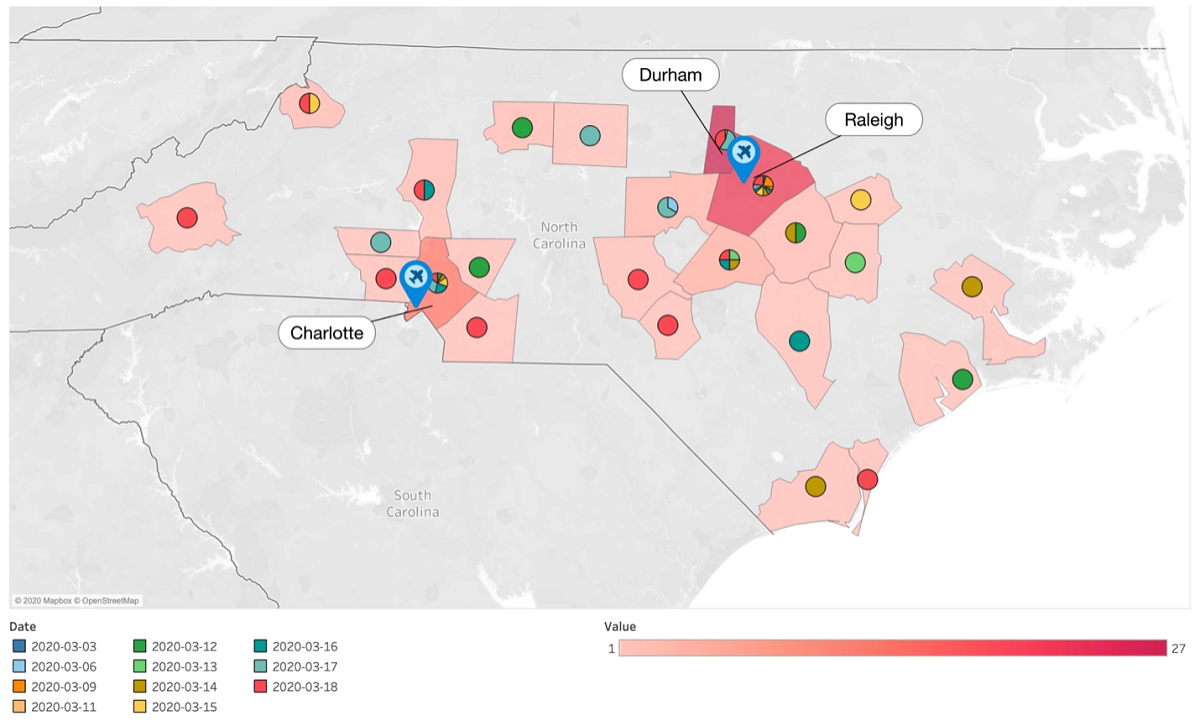
Time-motion analysis of confirmed coronavirus disease cases from March 3 to March 18, 2020.

Figure 2 shows the spread of confirmed COVID-19 cases by the day from March 3 to 18, 2020. The first confirmed case occurred on March 3, 2020, in Wake County, and the person had been travelling to Washington state and was exposed to a long-term facility where there was a COVID-19 outbreak [12]. The second case occurred on March 6, 2020 in Chatham County to a person who had returned from Italy where there had been a severe COVID-19 outbreak [13]. Chatham county is a neighboring county to Wake county where the RDU resides. The time-motion analysis shows that the first COVID-19 cases occurred in Wake county, while the highest prevalence of COVID-19 cases were in Wake county and Mecklenburg County. These two counties have two characteristics in common: they are the most populous and the only counties with an operating international airport. The color palettes in Figure 2 show that the disease systematically and quickly spread to the immediate neighboring counties in a matter of 3-5 days, and then systematically reached more distant counties in a relatively longer time span of 12-14 days.

### Analysis of Virtual Care Visits

**Figure 3:**
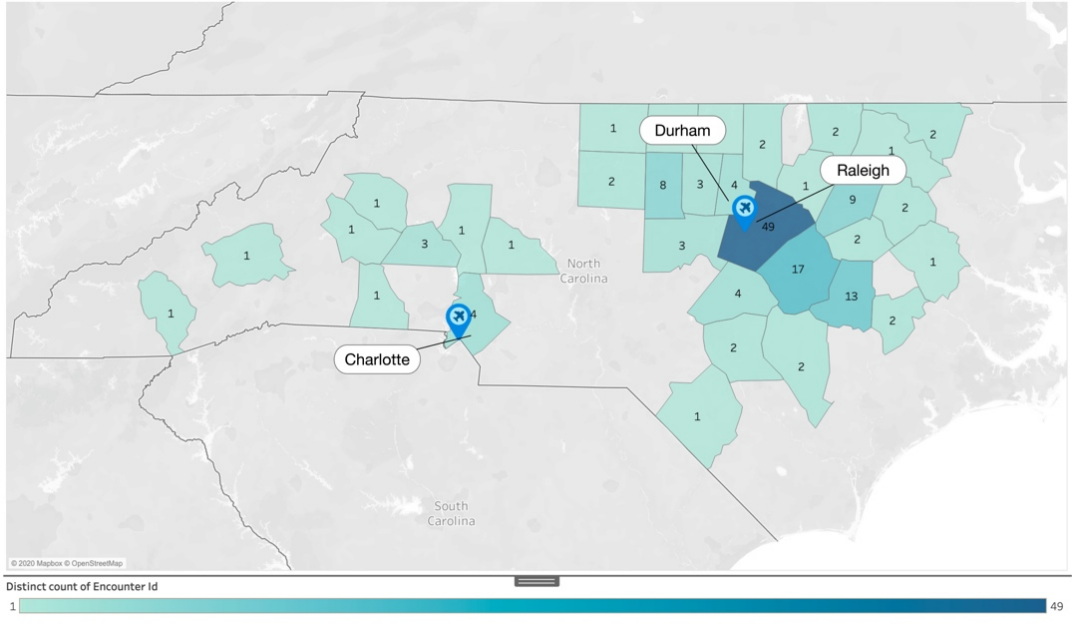
Quantification of virtual care visits from February 1 to 28, 2020.

We reported that 57.3% of COVID-19-like visits in February, prior to any confirmed cases, were initiated by individuals residing in the same high-density counties that later had confirmed cases. Of the 92 confirmed COVID-19 cases, Wake County, where RDU resides, had 49 (53%) visits, while Mecklenburg County, home of CLT, had 4 (4.3%) COVID-19-like visits (Figure 3). Additionally, during the first 12 days after the first confirmed COVID-19 case on March 3, the number of virtual visits related to COVID-19 in Wake and Mecklenburg county was 23 (24%) and 5 (5%), respectively.

## Discussion

### Principal Findings

We aimed to understand the trends in confirmed COVID-19 transmission and the use of VC to triage COVID-19-like symptoms. We found that a relationship exists between the geographic location of the initial confirmed COVID-19 cases with the population density and the presence of a functioning international airport. The first two cases in North Carolina came from individuals who were travelling back from areas with a COVID-19 outbreak. The spread of cases quickly transitioned into the immediate neighboring counties and then, further into more distant counties.

When looking at the number of VC COVID-19-like visits in the weeks prior to confirmed cases, we report that most of the COVID-19-like visits came from the same counties that later had confirmed cases. This has two interpretations. First, VC can help reduce the number of emergency department (ED) visits by providing remote medical consultation to patients residing in counties with increasing numbers of confirmed COVID-19 cases, which will reduce medical facilities overcrowding and thereby, control the spread of the disease. Second, it is possible that we can forecast the spread of the disease by monitoring the volume and location of confirmed COVID-19 cases, and the volume and location of visits in the VC realm, as shown in the time-motion study. Alternatively, the possibility of higher numbers of confirmed cases may lead to a higher number of virtual visits as individuals self-quarantine or exercise physical distancing.

This study provides several recommendations. First, limiting the movement of people through education and awareness of the importance of physical distancing and minimizing domestic and international travel, unless for emergencies, will help flatten the COVID-19 curve. Most of the new cases originated from people traveling to areas with active COVID-19 cases or in areas with high-population density where the probability of disease transmission is high.

Second, wider adoption and promotion of VC will reduce the number of unnecessary ED and urgent care visits, which is important during this time to avoid exhausting our health system and overcrowding, which increases the risk for transmission of the disease. 

Third, we need to use artificial intelligence and geospatial analytics to monitor and predict COVID-19 spread to better understand the trends in transmission, predict possible infected areas and the rate of transmission, and manage our workforce expectations [14-16]. This aligns with other calls for more research investigating the transmission of the virus and identifying vulnerable populations and regions [14].

The Centers for Medicare and Medicaid and insurance companies have waived telehealth restrictions in fear of exhausting our health care system capacity and resources, which should drive the push for more virtual-based case interventions. Although major health care systems are launching VC clinics, there seems to be a need for more promotion, especially among vulnerable and older populations who may not have the technological means to access such a service. We suggest continued efforts to deploy and promote the importance of VC as an important medium, as we fight for our existence.

### Study Limitations and Future Directions

This study presents data from a single health care system in one US state. The definition of COVID-19-like virtual visits was if the COVID-19 symptoms defined by WHO matched the chief complaints of the patient. Due to the lack of virtual COVID-19 screening and the rapid turn of events, we could not ensure that all these visits were actual COVID-19 cases.

Our future work will include an analysis of VC accessibility pre- and post-COVID-19 with regard to specific geographic locations. We are also interested in assessing the patient experience using VC during the COVID-19 period. Finally, we aim to evaluate the effectiveness of the telehealth expansion on in-person and virtual clinics. 

### Conclusions

The use of VC presents promising potential in the fight against COVID-19. VC can reduce emergency room visits, conserving health care resources, and avoid the spread of COVID-19 by treating patients remotely. We confirm that the largest spread of COVID-19 cases occurs in areas with a high population density as well as in areas with operating international airports. Our study also demonstrates that virtual care can provide efficient triaging in the counties with the highest number of COVID-19 cases. We call for speedier adoption of virtual care by health systems across US and the world during the COVID-19 pandemic.

## Abbreviations

CLT: Charlotte Douglas International Airport
COVID-19: coronavirus disease
ED: emergency department
NCDHHS: North Carolina Department of Health and Human Services
RDU: Raleigh-Durham International Airport
VC: virtual care
VUC: virtual urgent care
WHO: World Health Organization
